# Effect of variable antidiabetic treatments strategy on oxidative stress markers in obese patients with T2DM

**DOI:** 10.1186/s13098-017-0220-6

**Published:** 2017-05-25

**Authors:** Abeer A. ALrefai, Alsayed M. Alsalamony, Sameer H. Fatani, Hala F. M. Kamel

**Affiliations:** 1 Medical Biochemistry Department, Faculty of Medicine, Menoufia University, Shebîn El Kôm, Egypt; 2 Department of Internal Medicine, Diabetes and Endocrinology Unit, Mansoura University, Mansoura, Egypt; 3 Biochemistry Department, Faculty of Medicine, Umm Al-Qura University (UQU), Makka, Saudi Arabia; 4 Medical Biochemistry Department, Faculty of Medicine, Ain Shams University (ASU), Cairo, Egypt

**Keywords:** T2DM, Oxidative stress, OHA and insulin

## Abstract

**Aim:**

To evaluate the effect of different anti-diabetic treatment strategy on oxidative stress markers in patients with type 2 diabetes mellitus (T2DM).

**Subject and methods:**

A total of 93 patients with T2DM treated with metformin (G1 = 25), OHA (G2 = 22), OA and insulin (G3 = 26) and insulin alone (G4 = 20). In all patients, lipid profile and glycemic indices were assessed using routine laboratory tests. MDA and Oxidized LDL were assessed using commercially available ELISA kits. Laboratory tests were performed at baseline and at a control visit after 24 weeks of treatment.

**Results:**

A significant decrease in the levels of MDA with improvement of glycemic control was observed in the group receiving OHA in combination with insulin therapy. A similar decrease of oxLDL was observed in all diabetic subgroups with borderline significance in those receiving metformin alone. The remaining clinical and biochemical parameters were not changed during follow-up in any of the involved groups.

**Conclusion:**

A combination therapy with insulin was more effective in glycemic control and MDA reduction in T2DM. Whereas, a significant oxLDLc reduction was observed in T2DM irrespective of categories of antidiabetic treatment or glycemic control.

## Background

Diabetes mellitus (DM) is a major worldwide health problem and considered as one of the leading causes of death and disability [1], with estimated prevalence of more than 500 millions by 2035 [2]. Hyperglycemia, an important pathologic characteristic of type 2 diabetes mellitus (T2DM), measured by percentage of glycated hemoglobin (HbA1c) has long been linked to mortality associated with DM. Chronic hyperglycemia through polyol pathway and protein kinase C increase formation of reactive oxygen species (ROS) inducing a state of oxidative stress that has been proposed as a major pathophysiological link between progression of T2DM and the onset of diabetic complications [3]. Furthermore, oxygen free radical generation due to non-enzymatic protein glycosylation, aut-oxidation of glucose and changes in oxidant/antioxidant balance in DM [4]. Also, lipid alterations and lipoproteins oxidation have been also considered as contributory factors to oxidative stress in DM [5]. It has been shown that improvement in control is associated with reduced complications. However, intensive therapy to achieve near-normal HbA1c levels in patients with T2D has not been shown to reduce associated complications [6]. An effective interventions able of slowing progression of diabetes complications via alleviating oxidative stress are desirable. Despite, the critical role of antioxidants in diabetes, the clinical trials with conventional anti-oxidants and vitamins have either failed to exert beneficial effects or have inconsistent results [7]. Recently the role of of anti diabetic agents in oxidative stress reduction has been evaluated with controversial results. Thus, our study evaluated the role of different anti-diabetic strategy on oxidative stress markers and their relations with glycemic control, variable clinical and biochemical parameters.

## Methods

This prospective study was performed in 2015 at the outpatient clinic of Diabetes and Endocrinology Center—AL-Noor hospital-Makka. A total of 93 patients with T2DM were selected according to the following inclusion criteria; age >30 years, duration of diabetes more than 5 years. Based on their anti-diabetic strategy they were sub-classified into four groups; (G1) 25 (26.9%) (13 male and 12 female) treated by metformin, (G2) 22 (23.7%) (10 male and 12 female) received oral hypoglycemic agents (OHA) (A combination of metformin and sulfonylurea), (G3) 26 (28%) (16 male and 10 female) were treated by OHA and insulin and (G4) 20 (21.5%) (11 male and 9 female) were controlled with insulin alone. The decision-making for treatment based on glycemic control and patient compensation for treatment. The four groups were matched for age and gender. Pregnant or lactating female and patients with renal impairment (based on the value of estimated glomerular filtration rate of <60 ml/min according to the Modification of Diet in Renal Disease formula), other endocrine abnormalities, chronic diseases or other inflammatory disorders were also excluded from the study. This study was conducted according to the Declaration of Helsinki guidelines and was approved by the local ethical committee of Faculty of Medicine UQU and AL-Noor hospital. A written informed consent was signed by all participants. In order to evaluate the possible role of variable antidiabetic strategies in oxidative stress, the participants were followed for 24 weeks; the follow up visits were scheduled every 12 weeks of treatment. A full through history (soci-demographic, medical history) and clinical data were taken from all subjects. A venous blood samples were collected (in plain and EDTA tubes) from all participants after an over-night fasting and 2-h post prandial for analysis of glucose panel [fasting plasma glucose (FBG) and a 2-h post prandial glucose (2HPPG)], lipid profile [Total Cholesterol (TC), Triglycerides (TG), Low Density Lipoprotein Cholesterol (LDLc), High Density Lipoprotein Cholesterol (HDLc) and LDLc/HDLc], renal function (serum creatinine and blood urea), oxidative stress markers [malondialdehyde (MDA) and oxidized-Low Density Lipoprotein Cholesterol (ox-LDLc)] and inflammatory marker C-reactive protein (CRP). Also, glycemic control was measured by (HbA1c) (in whole blood) using a high-performance liquid chromatography. Serum Malondialdehyde (MDA) was assessed utilizing Quantichrom TBARS Assay Kit (DTBA-100) according to manufactures instructions. Serum ox-LDLc was analyzed using Enzyme-Linked-Immunosorbent Assay (ELISA) Cloud-Cron Corp Kit according to manufacturer’s instructions [8]. The diagnosis of DM is defined as a (FBG) ≥ 126 mg/dl, a (2HPPG) ≥ 200 mg/dl, HbA1C ≥ 6.5%, in a patient with classic symptoms of hyperglycemia [9]. Hypertension was diagnosed based on [systolic blood pressure (SBP) ≥ 140 mmHg and/or diastolic blood pressure (DBP) ≥ 90 mmHg in at least two separate measurements or regular use of anti-hypertensive medication] [10].

## Statistical analysis

The collected data were analyzed by statistical package for the social science (SPSS) software, version 16, Echosoft Corporation, USA. Categorical data are presented as percentages and continuous variables as mean ± SD for parametric data and median with range for non-parametric data. Comparisons between groups were calculated by Chi Square test with P for categorical variables and by *t* test and Mann–Whitney U test for continuous variables. The one-way analysis of variance (ANOVA), post hoc test and Kruskal–Wallis test were used when comparing more than two continuous variables. Spearman rank correlation analysis was used to determine associations between oxidative and inflammatory markers with selected parameters. Linear regression analysis was used to assess the independent predictors of oxLDLc. P values <0.05 were considered significant.

## Results

The baseline characteristics of the study participants are summarized in Tables 1 and 2. A total of 93 of patients with T2DM [G1 (52% male and 48% female), G2 (45.5% male and 54.5% female), G3 (61.5% male and 38.5% female) and G4 (55% male and 45% female)] were enrolled. The mean of their age and body mass index (BMI) were [(51.72 ± 5.8), (49.13 ± 9.11), (49.84 ± 3.7), (51.75 ± 4.15)], [(34.4 ± 6.41), (33.35 ± 5.59), (32.24 ± 3.58), (31.19 ± 3.41)] kg/m^2^, respectively. The study groups of diabetic patients were comparable for age (P = 0.35), gender (P = 0.73) and smoking habits (P = 0.22) with a similar distribution of BMI categories (P = 0.74). Those categorized in G4 suffered from DM for 10 (2–24) years (long duration) than the other subgroups (P = 0. 001) (Table 1). The median SBP and DBP were at pre-hypertensive level in G1 and G3, while G2 and G4 were hypertensive with significantly increased prevalence (59.1%) (70%) of HTN compared to G1(20%) and G3 (42.3%) (P = 0.004). Also G4 revealed a higher prevalence of neuropathy (P = 0.008) with increased inflammatory marker CRP in both G3 and G4 compared to G1 and G2 (P = 0.0001) (Table 1).

**Table 1:** Comparison of demographic and clinical data among studied groups

Variables	Group 1 (25)	Group 2 (22)	Group 3 (26)	Group 4 (20)	P value
Age (years)					0.35
X ± SD	51.72 ± 5.8	49.13 ± 9.11	49.84 ± 3.7	51.75 ± 4.15	
Gender					
Male	13 (52%)	10 (45.5%)	16 (61.5%)	11 (55%)	0.73
Female	12 (48%)	12 (54.5%)	10 (38.5%)	9 (45%)	
Duration (years)					0.001*
Median (range)	3 (1–10)	3 (1–11)	4 (1–24)	10 (2–24)	
Smoking	1 (4%)	1 (4.5%)	5 (19.2%)	3 (15%)	0.22
BMI category					
18–25	1 (4%)				
>25–30	5 (20%)	7 (31.8%)	6 (23.1%)	5 (25%)	0.74
>30	19 (76%)	15 (68.2%)	20 (76.9%)	15 (75%)	
SBP (mmHg)					0.41
X ± SD	138.8 ± 26.42	149.09 ± 26.48	139.62 ± 26.34	146.5 ± 17.45	
Range	130 (110–200)	152.5 (110–200)	127.5 (110–180)	150 (120–170)	
DBP (mmHg)					0.13
X ± SD	86.8 ± 10.09	91.13 ± 7.54	87.3 ± 8.74	91.75 ± 8.15	
Range	85 (70–120)	95 (80–100)	85 (75–100)	95 (80–100)	
HTN	5 (20%)	13 (59.1%)	11 (42.3%)	14 (70%)	0.004
IHD	2 (8%)	4 (18.2%)	2 (7.7%)	3 (15%)	0.61
HCT	2 (8%)	4 (18.2%)	6 (23.1%)	5 (25%)	0.43
Neuropathy	0	1 (4.5%)	6 (23.1%)	6 (30%)	0.008
CRP (mg/l) X ± SD	9.6 ± 3	13.41 ± 4.59	14.35 ± 1.54	19.8 ± 2.59	0.0001^◊#^

* Post hoc test significance between G4 vs. G1, G2 and G3

^◊^ Post hoc significance G3 and G4 vs. G1 and G2

^#^ Post hoc significance G1 vs. G2

**Table 2:** Character of the study groups regarding, anthropometric, routine biochemical data, inflammatory and oxidative stress markers at the 1 s visit

Variables	Group 1	Group2	Group 3	Group 4	P value
BMI kg/m^2^	34.4 ± 6.41	33.35 ± 5.59	32.24 ± 3.58	31.19 ± 3.41	0.16
FBG mg/dl	132.28 ± 21.57	131.59 ± 26.65	171.08 ± 25.71	179.6 ± 66.04	0.0001^◊^
	126 (73)	111 (100)	151 (59)	144 (172)	
PPBG mg/dl	166.36 ± 45.95	192.91 ± 37.23	200.13 ± 8.05	278.95 ± 15.1	0.0001^◊^
	155 (83–260)	194 (140–285)	201 (178–244)	290 (250–360)	
HbA1C%	6.83 ± 1.24	7.98 ± 1.17	8.49 ± 1.26	9.67 ± 1.64	0.0001^◊#^
TC mg/dl	174.84 ± 25.46	183.5 ± 35.39	172.6 ± 34.48	176.85 ± 44.97	0.74
TG mg/dl	148.64 ± 60.06	159.86 ± 53.83	168.77 ± 75.1	219 ± 93.28	
	136 (90–290)	143.5 (99–296)	140 (112–400)	208.5 (111–340)	0.01*
LDLc mg/dl	103.08 ± 23.37	102.314 ± 28	110.65 ± 29.32	106.8 ± 22.27	0.65
HDLc	43.8 ± 10.87	42.68 ± 8.81	40.654 ± 5.72	35.05 ± 7.29	0.005*
	41 (28–70)	38 (33–67)	40 (30–50)	32.5 (29–53)	
LDLc/HDLc	2.49 ± 0.83	2.51 ± 0.91	2.89 ± 1.31	3.21 ± 1.02	*0.09*
	3.11 (1.21–4.04)	2.53 (1.34–4.97)	2.83 (1.48–6)	3.28 (1.64–4.28)	
s.creatinine mg/dl	0.95 ± 0.04	0.95 ± 0.07	0.97 ± 0.07	0.94 ± 0.04	*0.58*
	0.9 (0.84–1.05)	0.97 (0.84–1.09)	0.98 (0.85–1.08)	0.94 (0.85–1)	
Urea mg/dl	28.76 ± 3.45	27.81 ± 2.38	27.92 ± 1.67	28.35 ± 2.1	0.55
MDA μM	9.62 ± 2.57	8.05 ± 2.63	10.38 ± 2.65	9.6 ± 3.68	*0.026*
	9 (5–12)	8.5 (4–13)	9 (7–12)	8 (7–14)	
OxLDLc pg/ml	1272.5 ± 395.35	1558.3 ± 552.7	1590.3 ± 586.57	1518.4 ± 292.61	
	1090 (600–2600)	1122 (625–2740)	1315 (670–2550)	1156 (880–2505)	*0.031*
OxLDLc/LDLc	11.47 ± 3.02	15.28 ± 7.44	14.76 ± 5.95	15.09 ± 4.92	*0.069*
	10.9 (6.7–18.4)	14.4 (5.4–37.5)	13.1 (6.5–25)	15.5 (9.1–24.2)	
OxLDLc/HDLc	31.38 ± 11.61	37.49 ± 13.69	39.81 ± 15.42	44.13 ± 11.99	*0.002*
	26.1 (16.8–62.3)	32.5 (23–73.2)	38.7 (15.9–67.2)	38.8 (32.5–66.3)	

Italic values indicate analysis of non-parametric data of more than two groups by Kruskal–Wallis test

^◊^ Post hoc significance G3 and G4 vs. G1 and G2

^#^ Post hoc significance G1 vs. G2

* Post hoc significance G4 vs. G1 and G2

Diabetic patients selected in G3 received insulin and OHA or G4 controlled by insulin alone initially differed from G1 and G2 received OHA by increased FPG of (171.08 ± 25.71, 179.6 ± 66.04 vs. 132.28 ± 21.57, 129.86 ± 28.73) mg/dl and 2H-PPG (166.36 ± 45.95, 192.91 ± 37.23, 200.23 ± 7.93, 279.15 ± 15.1) mg/dl (P = 0.0001). Also, there was an initial lack of metabolic control (HbA1C > 7.5%) in G2, G3 and G4 (7.91 ± 1.15, 8.59 ± 1.16, 9.67 ± 1.65 vs. 6.77 ± 1.18) compared to G1 (control group) (P = 0.0001). In addition, G4 patients revealed increased TG (P = 0.01) and decreased HDLc (P = 0.005) compared to those controlled by OHA (G1 and G2) (Table 2). However, patients with T2DM distributed in the different subgroups were matched for BMI (P = 0.16) (Table 2). Regarding oxidative stress markers both MDA, oxLDLc and oxLDL/HDLc initially revealed a significant difference among diabetic subgroups (P = 0.026), (P = 0.031) and (P = 0.002), mainly in G3 and both G3 and G4 respectively (Table 2). A significant decrease was observed in MDA [10.38 ± 2.65 to 7.34 ± 1.87 (P = 0.0001)] with improved glycemic control (HbA1C) [(8.49 ± 1.26 to 7.76 ± 0.93) during follow up at the 24th weeks mainly in G3 (Tables 3, 4). In addition, a significant reduction of the median oxLDLc [G2 (P = 0.002), G3 (P = 0.017) and G4 (P = 0.002)] and both oxLDL/LDLc and oxLDL/HDL [G2 (P = 0.009 & 0.001) and G4 (P = 0.018 & 0.002)] respectively was observed during follow up with a borderline significant reduction in G1 (P = 0.044) and G3 (P = 0.05) regarding oxLDLc and oxLDLc/HDLc respectively (Table 4). However, reduction of oxidative stress parameters [oxLDLc and both oxLDL/LDLc and oxLDL/HDL] not associated with an improved glycemic control. On the other hand, lipid profiles and BMI did not reveal any significant reduction during follow up, with no significant association with glycemic control. In addition, poorly controlled patients with T2DM revealed a high prevalence of neuropathy [(20% at 1st visit and 21.3% at last one) (P = 0.007 & 0.04)] regardless of antidiabetic strategy (not shown). The median ox-LDLc and MDA were significantly associated with hyperglycemia [PPG (r = 0.25, P = 0.015); 2hPPG (r = 0.3, P = 0.002)], diabetic complications [neuropathy (r = 0.25, P = 0.015) and inflammation (r = 0.26, P = 0.013) (Table 5). CRP an inflammatory marker was significantly associated with diabetes duration (r = 0.29, P = 0.004), hyperglycemia (r = 0.65, P = 0.0001), poor glycemic control (r = 0.39, P = 0.0001), and diabetes complications (Table 5). Moreover, regression analysis proved that hyperglycemia was independently associated with oxidative stress markers MDA [CI (0.015–0.048) P = 0.0001] (not shown) and oxLDLc [CI (0.53–4.44), P = 0.013] that displays a significant association with female gender, renal function [s.creatinine CI (204–534), P = 0.035] and hypertension [SBP CI (9–32), P = 0.0001] (Table 6).

**Table 3:** Character of the study groups regarding, anthropometric, routine biochemical data, inflammatory and oxidative stress markers at the end of the study

Variables	Group 1	Group 2	Group 3	Group 4	P value
BMI kg/m^2^	34.64 ± 7.07	32.97 ± 5.53	31.41 ± 3.73	30.73 ± 3.29	0.054
FBG mg/dl	127.8 ± 17.48	126.05 ± 23.31	150.58 ± 10.03	164.85 ± 47.51	0.0001^•^
	124 (78)	111.5 (96)	150 (60)	140 (151)	
PPBG mg/dl	157.2 ± 35.71	179.32 ± 30.01	182.27 ± 8.72	249.5 ± 15.71	0.0001^#•^
	153 (135)	189 (145)	200 (32)	282 (52)	
HbA1C%	6.77 ± 0.84	7.73 ± 1.17	7.76 ± 0.93	8.85 ± 1.72	0.0001^#”^
TC mg/dl	172.6 ± 25.42	182.41 ± 36.29	171.08 ± 35.02	174.5 ± 44.31	0.69
TG mg/dl	147.54 ± 59.01	157.66 ± 51.63	166.67 ± 73.01	217 ± 91.26	
	136 (90–290)	143.5 (99–296)	140 (112–400)	208.5 (111–340)	0.02*
LDLc mg/dl	99.28 ± 18.54	93.68 ± 26.66	112.42 ± 28.83	94.15 ± 19.86	0.027^◊^
HDLc	43.88 ± 9.47	41.09 ± 7.77	42.46 ± 4.39	32.85 ± 4.74	0.0001^#^
	40 (35)	40 (26)	43.5 (17)	32 (16)	
LDcL/HDLc	2.37 ± 0.67	2.38 ± 0.88	2.75 ± 1.11	2.92 ± 0.71	*0.048*
	22 (2.4)	2.13 (3.15)	2.47 (4.49)	2.73 (2.33)	
s.creatinine mg/dl	0.95 ± 0.04	0.95 ± 0.06	0.96 ± 0.06	0.94 ± 0.04	*0.56*
	0.9 (0.84–1.05)	0.97 (0.84–1.09)	0.98 (0.85–1.08)	0.94 (0.85–1)	
Urea mg/dl	28.70 ± 3.42	27.8 ± 2.36	27.9 ± 1.65	28.35 ± 2.1	0.53
MDA μM	8.4 ± 2.55	8.04 ± 2.72	7.34 ± 1.87	8.7 ± 2.58	*0.29*
	7.1 (8)	8 (10.8)	7 (5.5)	8.5 (7)	
OxLDLc pg/ml	1135.4 ± 427.5	1063.8 ± 513.64	1277.3 ± 590.4	1263.4 ± 1064.5	*0.22*
	980 (1976)	1011.5 (2030)	1065 (1916)	874 (3206)	
OxLDLc/LDLc	10.26 ± 3.83	10.16 ± 5.47	11.86 ± 5.55	13.48 ± 12.95	*0.54*
	9.77 (16.67)	9.17 (27.18)	11.83 (17.11)	8.84 (40.5)	
OxLDLc/HDLc	29.17 ± 16.86	26.32 ± 14.49	32.12 ± 15.91	37.86 ± 36.35	*0.39*
	23.08 (72.83)	21.52 (55.64)	28.05 (49.47)	21.85 (106.44)	

Italic values indicate analysis of non-parametric data of more than two groups by Kruskal–Wallis test

* Post hoc significance between G1 vs.G2, G3 and G4

^•^ Post hoc significance between G3 and G4 vs. G1 and G2

^#^ Post hoc significance between G4 vs. G1, G2 and G3

^“^ Post hoc significance between G1 vs. G2 and G3

^◊^ Post hoc significance between G3 vs. G2

**Table 4:** The course for clinical parameters in studied groups

Variables	Group 1	Group 2	Group 3	Group 4
HbA1C1	6.83 ± 1.24	7.98 ± 1.17	8.49 ± 1.26	9.67 ± 1.64
HbA1C3	6.77 ± 0.8	7.73 ± 1.17	7.76 ± 0.93	8.85 ± 1.72
Reduction in HbA1C	−0.06 ± 1.04	−0.25 ± 0.72	−0.73 ± 1.27	−0.82 ± 0.78
P value within group	P = *0.97*	P = *0.39*	P = *0.023*	P = *0.11*
OxLDLc1	1272.5 ± 395.35	1558.3 ± 552.74	1590.3 ± 586.57	1518.4 ± 292.61
OxLDLc3	1135.4 ± 427.51	1063.8 ± 513.64	1277.3 ± 590.41	1263.4 ± 1064.56
Reduction in oxLDLc	−137.1 ± 517.52	−494.5 ± 625.1	−312.9 ± 514.55	−255 ± 931.2
	−131(−1744–870)	−513.5(−2066–1255)	−391(−1100–833)	−639(−1379–1585)
P value within group	P = *0.044*	P = *0.002*	P = *0.017*	P = *0.002*
OxLDL/LDLc1	11.47 ± 3.02	15.28 ± 7.43	14.76 ± 5.95	15.09 ± 4.92
OxLDLc/LDLc3	10.26 ± 3.83	10.16±5.47	11.86 ± 5.55	13.48 ± 12.95
Reduction in oxLDLc/LDLc	−1.2 ± 4.08	−5.12 ± 5.03	−2.89 ± 4.53	−1.62 ± 9.92
	−1.38(−10.38–7.7)	−5.5(−13.11–7.22)	−2.34(−11.96–5.37)	−4.96(−11.83–19.33)
P value within group	P = *0.15*	P = *0.009*	P = *0.07*	P = *0.018*
OxLDL/HDLc1	31.38 ± 11.61	37.49 ± 13.69	39.81 ± 15.42	44.13 ± 11.99
OxLDL/HDLc3	29.17 ± 16.86	26.32 ± 14.49	32.12 ± 15.91	37.86 ± 36.36
Reduction in oxLDLc/HDLc	−2.21 ± 12.76	−11.18 ± 15.93	−7.68 ± 12.84	−6.28 ± 29.43
	−2.98(−32.3–30)	−12.88(−54.39–35.88)	−7.82(−25–22.51)	−15.98(−47.55–52.83)
P value within group	P = *0.11*	P = *0.001*	P = *0.05*	P = *0.002*
MDA1	9.62 ± 2.56	8.05 ± 2.63	10.38 ± 2.65	9.6 ± 3.68
MDA3	8.41 ± 2.55	8.04 ± 2.72	7.34 ± 1.87	8.7 ± 2.58
Reduction in MDA	−1.2 ± 3.34	−0.01 ± 3.37	−3.03 ± 2.95	−0.9 ± 3.55
	−1.2(−7–7)	−1.5 (−8–6)	−2(−9–0.8)	−0.5(−8–4)
P value within group	P = *0.1*	P = *0.93*	P = *0.0001*	P = *0.31*

Italic values indicate analysis of non-parametric data between two groups by Mann–Whitney test

**Table 5:** Correlation of oxidative stress and cardiac biomarkers with variable parameters

	OxLDLc1		MDA1		OxLDLc3		MDA3		CRP	
	r	P	r	P	r	P	r	P	r	P
Age	0.089	0.39	−0.13	0.22	0.15	0.15	0.17	*0.09*	0.03	0.77
Gender	0.1	0.034	0.04	0.69	−0.28	0.007	−0.009	0.94	−0.13	0.22
Smoking	−0.12	0.24	0.027	0.79	−0.16	0.12	0.08	0.45	−0.11	0.28
D/duration	0.09	0.42	−0.06	0.53	0.03	0.8	0.05	0.65	0.29	*0.004*
BMI	−0.25	0.014	0.15	0.15	0.21	0.049	−0.03	0.82	−0.23	*0.025*
Neuropath	0.25	*0.015*	−0.08	0.4	0.14	0.19	0.02	0.88	0.29	*0.004*
FBG	−0.007	0.95	0.3	*0.002*	0.01	0.92	−0.07	0.49	0.23	*0.025*
PPBG	0.25	*0.015*	0.16	0.13	−0.08	0.47	0.11	*0.28*	0.65	*0.000*
HbA1C	0.09	0.39	0.16	0.12	−0.16	*0.13*	0.16	0.12	0.39	*0.000*
TC	−0.014	0.39	−0.09	0.36	−0.06	0.58	0.07	0.49	−0.008	0.94
TG	0.1	0.33	0.03	0.75	−0.04	0.71	0.19	0.065	0.32	*0.002*
LDLc	0.03	0.79	0.15	0.15	0.12	0.24	0.001	0.99	0.07	0.52
HDLc	0.03	0.75	0.01	0.9	0.03	0.76	0.019	0.86	−0.25	*0.014*
LDLc/HDLc	0.13	0.9	0.06	0.56	0.08	0.44	−0.06	0.55	0.15	0.12
S.creatinin	0.17	0.11	−0.006	0.95	0.34	*0.001*	0.07	0.51	−0.03	0.79
Urea	−0.09	0.37	−0.2	0.053	0.23	*0.03*	0.23	0.025	0.06	0.57
CRP	0.26	*0.013*	0.04	0.7	0.02	0.85	0.19	0.06		

Italic values are statistically significant (P < 0.05)

**Table 6:** Linear regression analysis to investigate independent factors associated with oxLDL in diabetic patients

Variables	OxLDLc1			OxLDLc3		
	B	CI	P	B	CI	P
Smoking	−0.042	(−452–320.9)	0.74	0.024	(−609–505)	0.85
Gender	0.31	(103–490)	*0.003*	−0.13	(−458–103)	0.21
SBP	0.76	(7.2–22.9)	*0.0001*	0.78	(9–32)	*0.0001*
DBP	−0.79	(−65–21.8)	*0.0001*	−0.54	(−72–8)	*0.013*
Neuropathy	−0.76	(−493–279)	0.58	0.08	(−395–704)	0.58
FBG	−0.15	(−3.9–0.51)	0.13	−0.059	(−5.7–3.1)	0.57
PPG	0.26	(0.53–4.44)	*0.013*	0.12	(−1.5–5.3)	0.28
S. creatinine	0.4	(1487–5080)	*0.0001*	0.25	(204–534)	*0.035*

Predictors smoking, gender, SBP, DBP. NEUROPAHY, FBG, 2H-PPG and S. creatinine

Italic values indicate linear regression analysis

## Discussion

This study demonstrated the effect of variable anti-diabetic treatment strategy on the oxidative stress biomarkers regarding glycemic control and their effect on lipoprotein parameters in obese patients with T2DM. Silvares et al. have shown an elevated oxidative stress with inflammation, microvascular damage and AGEs deposition in HFD-/STZ-induced diabetes in rats [11]. Hyperglycemia induces ROS production, that initiate a chain reaction leading to an increased inflammatory response and chemical modification of lipoproteins [12]. Thus the profiles of the transported lipids in diabetes are characterized not only by their increased levels, but also by aberrant patterns [13]. Most previous studies detect induced oxidative stress in T2DM, represented through increased MDA, oxLDL and F2-Isoprostanes [14, 15]. Our results indicate that in T2DM the activation of oxidative stress appears to be influenced not only by hyperglycemia but also by the categories of anti-diabetic treatments. Monnier et al. observed that oxidative stress is more pronounced in patients receiving insulin alone compared to those receiving both OHA and insulin; indicating the role of hyperinsulinemia in exaggerating oxidative stress [16]. Thus the activation of oxidative stress could appear to depend on the categories of anti-diabetic treatments (OHA alone or in combination with insulin) and secondly on the total daily doses of insulin employed. In concern with their finding, we observed a significant reduction of oxidative stress (MDA) in those receiving OHA and insulin compared to those receiving insulin alone. Also, Zhang et al. reported that the 2-h PPG level was still at a higher level in insulin monotherapy, whether a combination therapy with insulin induces an improvement of lipid profile, body weight, blood pressure and MDA secretion [17]. On the other hand, Silvares et al. stated that insulin monotherapy and metformin adjunct treatment improved body weight, % HbA1c, and oxidative stress parameters similarly. Whether, the metformin adjunct treatment improved fasting blood glucose level than insulin monotherapy [11]. Additionally, Njajou et al. reported a strong linear association of HbA1c for MDA and oxLDL [18]. Our study, proved a significant reduction of MDA with improvement of glycemic control in those receiving insulin adjunct treatments. Moreover, a significant reduction of oxLDLc with no improved glycemic control was demonstrated in our study irrespective of anti-diabetic medication categories. On the other hand, Megson et al. demonstrated a significant reduction of ox-LDL with improved glucose control (reduced glucose excursions), but not with insulin dose [19]. However, Burchardt et al. observed a significant reduction of glycated LDL but not oxLDLc in those receiving insulin or combination of metformin to intensive insulin therapy [20]. In our study those receiving metformin alone revealed a non-significant or border-line significant reduction of MDA and oxLDLc respectively. In contrast, a recent study demonstrated that metformin treatment ameliorated high glucose-induced beta cell dysfunction by decreasing intracellular ROS production [21]. The contribution of metformin to oxidative stress inhibition could be explained by its anti-inflammatory role [22]. Forsberg et al. stated that the peripheral neuropathy was strongly associated with oxidative stress and inflammatory markers, confirming our results and proving the role of oxidative stress in driving progression of T2DM and in mediating complications associated with the disease [23], thus the modulation of oxidative stress represents an important target for therapeutic intervention. It was proved that severe oxidative stress in T2DM with increased BMI has been associated with hyperglycemia, glycemic control, insulin resistance and disease duration [24–26]. However, the positive association of oxLDLc with incident T2DM, was attenuated after adjustment for BMI [27]. The metformin therapy was preferred not only for its anti-hyperglycemic effect, but also for its weight-reducing and insulin resistance-decreasing properties. Adjunct metformin reduces the insulin dose requirement and stabilizes weight with potential impact on cardiovascular risk factors and complications [28]. Our study revealed non-significant reduction of BMI or postprandial lipids in comparison with base-line values regardless the type of anti-diabetic medication. This controversy could be due to lack of evaluation of associated life style change and short study duration. The different levels of FPG, and PPG at the baseline between the compared groups were the major confounding factor. Also, the received hypolipidemic pharmacotherapy that have been discussed previously to act in an antioxidative manner with DNA-damage-protecting properties, should be considered as another confounding factor [29].

## Conclusion

This study demonstrated that in T2DM the oxidative stress activation is influenced not only by hyperglycemia but also by the categories of anti-diabetic treatment. An OHA in combination with insulin therapy induce significant reduction of MDA with improvement of glycemic control, whereas a significant reduction of oxLDL (related to diabetic complications) was observed in diabetic patients irrespective of glycemic control or the categories of anti-diabetic treatment (whether they were treated with OHA alone, OHA and insulin or insulin alone). However, all the antidiabetic treatment strategy induces a non-significant reduction of BMI or lipid profiles. This suggests that the effect of combination therapy or insulin therapy alone on oxidative stress influenced by variable factors is complicated and a further large clinical randomized study is recommended.

## Abbreviations

2H-PPG: 2 h post prandial glucose
ANOVA: Analysis of variance test
BMI: body mass index
CRP: C-reactive protein
DM: diabetes mellitus
FBG: fasting blood glucose
HDLc: high density lipoprotein cholesterol
MDA: malondialdehyde
OHA: oral hypoglycemic agents
OxLDLc: oxidiazed low density lipoprotein cholesterol
ROS: reaactive oxygen species
SPSS: (statistical package for the social science) software
T2DM: type 2 diabetes mellitus
TBARS: thiobarbituric acid reactive substance
TC: total cholesterol
TG: triglycerides

## References

[1] Matough FA, Budin SB, Hamid ZA, Alwahaibi N, Mohamed J (2012). The role of oxidative stress and anti-oxidant in diabetes complications. Sultan Qaboos Univ Med J.

[2] Guariguata L, Whiting DR, Hambleton I, Beagley J, Linnenkamp U, Shaw JE (2014). Global estimates of diabetes prevalence for 2013 and projections for 2035. Diabetes Res Clin Pract.

[3] Yan M, Mehta JL, Zhang W, Hu C (2011). “LOX-1, oxidative stress and inflammation, a novel mechanism for diabetic cardiovascular complications. Cardiovasc Drugs Ther.

[4] Ahmed N (2005). Advanced glycation end products—role in pathology of diabetic complications. Diabetes Res Clin Pract.

[5] Vergès B (2005). New insight into the pathophysiology of lipid abnormalities in type 2 diabetes. Diabetes Metab.

[6] Gerstein HC, Miller ME, Byington RP (2008). Effects of intensive glucose lowering in type 2 diabetes. New Engl J Med.

[7] Pazdro R, Burgess J (2010). The role of vitamin E and oxidative stress in diabetes complications. Mech Aging Dev.

[8] Fan A, Wu X, Wu H, Li L, Huang R, Zhu Y, Qiu Y, Fu J, Ren J, Zhu C (2014). Atheroprotective effect of oleoylethanolamide (OEA) targeting oxidized LDL. PLoS One.

[9] American Diabetes Association (2015). Standards of medical care in diabetes-2015 abridged for primary care providers. Clin Diabetes.

[10] Chobanian AV, Bakris GL, Black HR, Cushman WC, Green LA, Izzo JL (2003). National high blood pressure education program coordinating, C. seventh report of the Joint National Committee on prevention, detection, evaluation, and treatment of high blood pressure. Hypertension.

[11] Silvares RR, Pereira EN, Flores EE, Estato V, Reis PA, Silva IJ, Machado MP, Neto HC, Tibiriça E, Daliry A (2016). Combined therapy with metformin and insulin attenuates systemic and hepatic alterations in a model of high-fat diet-/streptozotocin-induced diabetes. Int J Exp Pathol.

[12] Wright E, Scism-Bacon JL, Glass LC (2006). Oxidative stress in type 2 diabetes: the role of fasting and postprandial glycaemia. Int J Clin Pract.

[13] Burchardt P, Zawada A, Wierusz-Wysocka B (2012). Cardiovascular risk associated with abnormal metabolism of plasma lipoproteins in patients with diabetes mellitus. Kardiol Polska.

[14] Aouacheri O, Saka S, Krim M, Messaadia A, Maidi I (2015). The investigation of the oxidative stress-related parameters in type2 diabetes mellitus. Can J Diabetes.

[15] Grindel A, Guggenberger B, Eichberger L, Pöppelmeyer C, Gschaider M, Tosevska A (2016). Oxidative stress, DNA damage and DNA repair in female patients with diabetes mellitus type2. PLoS ONE.

[16] Monnier L, Colette C, Michel F, Cristol JP, Owens DR (2011). Insulin therapy has a complex relationship with measure of oxidative stress in type2 diabetes: a case for further study. Diabetes Metab Res Rev.

[17] Zhang X, Liu Y, Xiong D, Xie C (2015). Insulin combined with Chinese medicine improves glycemic outcome through multiple pathways in patients with type 2 diabetes mellitus. J Diabetes Investig.

[18] NjajouO T, Kanaya AM, Holvoet P, Connelly S, Strotmeyer ES, Harris TB (2009). Association between oxidized LDL, obesity and type2 diabetes in a population-based cohort, the health, aging and body composition study. Diabetes Metab Res Rev.

[19] Megson IL, Treweeke AT, Shaw A, MacRury SM, Setford S, Frias JP, Anhalt H (2015). Continuous subcutaneous insulin infusion in patients with type 2 diabetes: a cohort study to establish the relationship between glucose control and plasma oxidized low density lipoprotein. J Diabetes Sci Technol.

[20] Burchardt P, Zawada A, Tabaczewski P, Naskręt D, Kaczmarek J, Marcinkaniec J, Wierusz-Wysocka B, Wysocki H (2013). Metformin added to intensive insulin therapy reduces plasma levels of glycated but not oxidized low-density lipoprotein in young patients with type 1 diabetes and obesity in comparison with insulin alone: a pilot study. Pol Arch Med Wewn.

[21] Moon JS, Karunakaran U, Elumalai S, Lee I-K, Lee HW, Kim YW, Won KC (2016). Metformin prevents glucotoxicity by alleviating oxidative and ER stress–induced CD36 expression in pancreatic beta cells. J Diabetes Complicat.

[22] Isoda K, Youg J, Kirilke A, Macfralane LA, Tsuobi N, Gerdes N (2006). Metformin inhibits pro-inflammatory response and nuclear factor-KB in human vascular wall cells. Arterioscler Thromb Vasc Biol.

[23] Forsberg E, Xu C, Grunler J, Frostgard J, Tekle M, Brismar K, Karvested L (2015). Coenzyme Q10 and oxidative stress, the association with peripheral sensory neuropathy and CVD IN T2DM. J Diabetes Complicat.

[24] Reddy VS, Agrawal P, Sethi S, Gupta N, Garg R, Madaan H, Kumar V (2015). Associations of FPG, A1C and disease duration with protein markers of oxidative damage and antioxidative defense in type 2 diabetes and diabetic retinopathy. Eye.

[25] Tangvarasittichai S, Pongthaisong S, Tangvarasittichai O (2016). Tumor necrosis factor-Α, interleukin-6, C-reactive protein levels and insulin resistance associated with type 2 diabetes in abdominal obesity women. Indian J Clin Biochem.

[26] Kang HM, Kim DJ (2012). Body mass index and waist circumference according to glucose tolerance status in Korea. The 2005 Korean health and nutrition examination survey. J Korean Med Sci.

[27] Odegaard AO, Jacobs DR, Sanchez OA, Goff DC, Reiner AP, Gross MD (2016). Oxidative stress, inflammation, endothelial dysfunction and incidence of type 2 diabetes. Cardiovasc Diabetol.

[28] Petrie JR, Chaturvedi N, Ford I, Hramiak I, Hughes AD, Jenkins AJ, Klein BE, Klein R, Ooi TC, Rossing P, Sattar N, Stehouwer CDA, Colhoun HM (2017). Metformin in adults with type 1 diabetes: design and methods of REducing with MetfOrmin vascular adverse lesions (REMOVAL): an international multicentre trial. Diabetes Obes Metab.

[29] Schupp N, Schmid U, Heidland A, Stopper H (2008). Rosuvastatin protects against oxidative stress and DNA damage in vitro via upregulation of glutathione synthesis. Atherosclerosis.

